# Obstacle Feature Information-Based Motion Decision-Making Method for Obstacle-Crossing Motions in Lower Limb Exoskeleton Robots

**DOI:** 10.3390/biomimetics10050311

**Published:** 2025-05-12

**Authors:** Yuepeng Zhang, Guangzhong Cao, Jun Wu, Bo Gao, Linzhong Xia, Chen Lu, Hui Wang

**Affiliations:** 1 School of Sino-German Robotics, Shenzhen Institute of Information Technology, Shenzhen 518172, China; ypzhang@sziit.edu.cn (Y.Z.); wujun@sziit.edu.cn (J.W.);gaob@sziit.edu.cn (B.G.); xialz@sziit.edu.cn (L.X.); 2 Inovance Industrial Robot Reliability Technology Research Institute, Shenzhen Institute of Information Technology, Shenzhen 518172, China; luchen@sziit.edu.cn (C.L.); wanghui@sziit.edu.cn (H.W.); 3 Guangdong Key Laboratory of Electromagnetic Control and Intelligent Robots, Shenzhen University, Shenzhen 518060, China

**Keywords:** lower limb exoskeleton robot, obstacle-crossing motion, gait trajectory planning

## Abstract

To overcome the problem of insufficient adaptability to the motion environment of lower limb exoskeleton robots, this paper introduces computer vision technology into the motion control of lower limb exoskeleton robots and studies an obstacle-crossing-motion method based on detecting obstacle feature information. Considering the feature information of different obstacles and the distance between obstacles and robots, a trajectory planning method based on direct point matching was used to generate offline adjusted gait trajectory libraries and obstacle-crossing gait trajectory libraries. A lower limb exoskeleton robot obstacle-crossing motion decision-making algorithm based on obstacle feature information is proposed by combining gait constraints and motion constraints, enabling it to select appropriate motion trajectories in the trajectory library. The proposed obstacle-crossing-motion method was validated at three distances between the obstacle and the robot and with the feature information of four obstacles. The experimental results show that the proposed method can select appropriate trajectories from the trajectory library based on the detected obstacle feature information and can safely complete obstacle-crossing motions.

## 1. Introduction

The brain of individuals with sound physical abilities can perceive the movement environment and their state through visual reflex nerves and make corresponding decisions and exercise behaviors based on environmental factors that affect movements [1,2]. However, people with lower limb disabilities cannot execute their brain’s intentions smoothly, requiring external assistive devices to complete the desired actions [3,4]. In existing research, there is a potential for lower limb exoskeleton robots to perform desired human movements through brain–computer interfaces [5,6,7,8]. However, EEG signals are extremely weak [9], and there are challenges in applying brain–computer interfaces for motion control of lower limb exoskeleton robots [10,11]. Computer vision technology has significant advantages in the perception of motion environment for lower limb exoskeleton robots [12,13,14,15]. Using depth cameras or visual sensors [16,17,18,19,20,21] to accurately obtain obstacle feature information and distance, and then converting it into executable instructions to transmit to the controller of the lower limb exoskeleton robot, it can make scientific motion decisions based on the detected obstacle-related information, achieving safe obstacle-avoidance motion and avoiding collisions and falls [22].

At present, there are few reports on obstacle avoidance based on computer vision for lower limb exoskeleton robots. Laschowski et al. [14] designed an environment recognition system based on computer vision and deep learning. In this system, an optical camera is worn on the chest to record environmental data, and these environmental data are integrated into the controller of the lower limb exoskeleton robot. The assistive motions of the lower limb exoskeleton robot are achieved by combining with feature data, such as IMU and goniometer data. Liu et al. [17] developed a visual-assisted autonomous gait planning method to improve the adaptability of lower limb exoskeleton robots to their motion environment. They obtained environmental information through an RGB-D camera and extracted ground object information that affects gait. Based on environmental information, the robot status, and safety constraints, they planned the gait pattern of the lower limb exoskeleton robot that can cross obstacles. Hua et al. [18] proposed a visual information-based obstacle-clearance strategy to enhance the exoskeleton system’s ability to navigate obstacles. Their study designed a hybrid boundary box strategy that combines proximity regression in L-shaped sections and synchronous convergence in a convex hull search to accurately obtain the cross-sectional, attitude, and size characteristics of multiple obstacles in space. Their study also established a switching criterion based on the principle of instantaneous capture point transfer and inflection point guided interpolation to achieve humanoid behavior in the gait optimization process, to realize nonlinear model predictive control, and to avoid obstacles in the environment. Ramanathan et al. [23] studied visual-based environmental perception technology aimed at enabling lower limb exoskeleton robots to detect and cross obstacles. This study proposes a similarity measurement method based on color, gradient direction, and two-dimensional surface normal vectors to distinguish noise caused by the perception errors of obstacles and uses obstacle depth image data combined with inertial measurement unit data to obtain obstacle-related distances and sizes. The research results indicate that the proposed visual environment perception technology can enable lower limb exoskeleton robots to understand the environment and cross obstacles. Trombin et al. [24] proposed a new environment-adaptive gait planning method to solve the obstacle-avoidance problem of lower limb exoskeleton robots when walking in complex environments. This study uses RGB-D cameras combined with a random sampling consensus algorithm to identify the ground and obstacles and their relative positions. The authors use forward kinematics to calculate the pose of the robot in the environment and then use a Gaussian iterative search algorithm to obtain gait parameters to satisfy kinematic and safety constraints, thereby generating collision-free foot trajectories. However, this study only considered single-step planning and did not consider multi-step or continuous walking.

Some researchers have integrated two or more sensors to improve the environmental perception ability of lower limb exoskeleton robots. Wang et al. [25] developed a multi-sensor unit that integrates three wide-angle cameras and RGB-D sensors, providing precise environmental perception capabilities for exoskeletons. This study utilizes a language-extended indoor SLAM modeling environment, generates high-precision topographic maps by fusing RGB-D data, and analyzes the accessibility of the terrain. The experimental results show that the proposed method can effectively evaluate terrain accessibility, with an accurate F-score of 0.93. Wu et al. [26] integrated Hololens and Realsense devices to construct a perception and interaction platform based on mixed reality and stereo vision and proposed a visual gait planning system for lower limb exoskeleton robots in complex terrains. This study used the Bessel curve for path planning and used heuristic search algorithms to search for the optimal gait path within the restricted area. The results indicate that the proposed method can plan smooth and feasible gait paths in complex terrains. Tricomi et al. [27] studied hip exoskeleton-assisted movements using computer vision technology. Using an RGB camera to capture the type of environment in front of the motion (stairs or flat ground), an adaptive oscillator estimates the user’s gait phase from IMU data, and a modulation strategy adjusts the assist level of each leg to adapt to environmental changes. Although the control strategy proposed in this study performed well in experiments, the applicability of this method needs further verification.

Although the above research achieved obstacle crossing for lower limb exoskeleton robots, it did not consider adjustments of the gait amplitude and decision-making for obstacle crossing for different obstacles and distances. This is important for improving the environmental adaptability of lower limb exoskeleton robots. In addition, although some studies have integrated multiple visual auxiliary sensors to aid in environmental perception, the operation of multiple sensors requires high computing power, which poses a challenge to the computing resources of lower limb exoskeleton robot systems.

The challenge of obstacle-crossing motions for lower limb exoskeleton robots involves making scientific decisions about movements based on obstacle feature information to ensure safe navigation. To tackle this issue, this paper integrates computer vision technology with the motion control of lower limb exoskeleton robots, establishing two offline gait trajectory libraries through trajectory planning methods. An obstacle-crossing-motion decision-making algorithm, which depends on obstacle feature information, combines motion and gait constraints within human–robot systems. The proposed algorithm selects appropriate robot motion trajectories from the trajectory library based on the detected obstacle features to promote safe obstacle-crossing motions.

## 2. Method of Obstacle-Crossing Motions

### 2.1. Acquisition of Obstacle Feature Information

To accurately obtain obstacle information in the motion environment of lower limb exoskeleton robots, the depth camera must be calibrated to obtain its internal parameters [28].

(1) $$K=\left(\begin{array}{ccc} f/d_{x} & 0 & n_{xo}\\ 0 & f/d_{y} & n_{yo}\\ 0 & 0 & 1 \end{array}\right)$$

where f is the focal length of the depth camera, and $d_{x},d_{y},n_{xo},n_{yo}$ are the dimensions of the image in the *x* and *y* directions and the center of the image in the *x* and *y* directions, respectively.

The two-dimensional environmental depth map obtained by the depth camera is converted into a three-dimensional point cloud to facilitate the recognition of environmental information. $n_{x}$ and $n_{y}$ are the *X*-axis and *Y*-axis of the image coordinate system, and $\left(P_{X},P_{Y},P_{Z}\right)$ are the coordinates of the target point. $P_{Z}$ is the value of the target point and the z-axis direction of the depth camera. Therefore, the coordinates of the target point are as follows [17]:

(2) $$\left[\begin{array}{c} P_{X}\\ P_{Y}\\ P_{Z} \end{array}\right]=P_{Z}{\left[\begin{array}{ccc} f/d_{x} & 0 & n_{xo}\\ 0 & f/d_{y} & n_{yo}\\ 0 & 0 & 1 \end{array}\right]}^{-1}\left[\begin{array}{c} n_{x}\\ n_{y}\\ 1 \end{array}\right]$$

The coordinate values of object surface sampling points obtained through depth camera scanning in the world coordinate system are used to obtain the feature information of obstacles in the world coordinate system.

Before conducting obstacle detection, the first step is to obtain ground information. The ground is defined as an ideal plane, which can be represented in a three-dimensional space as

(3) Xx+Yy+Zz=P

where $\left(x,y,z\right)$ is the point on the spatial fitting plane; $\left(X,Y,Z\right)$ is the unit normal vector of the plane; and P is the distance from the coordinate origin to the plane.

The key to extracting ground information from three-dimensional point cloud data is to determine X, Y, Z and P. If the point cloud dataset is defined as $D_{O}=\left(x_{i},y_{i},z_{i}\right){|}_{i=1}^{N}$, then the points on the plane need to satisfy

(4) $$\left[\begin{array}{cc} D_{O} & -1 \end{array}\right]{\left[\begin{array}{cccc} X & Y & Z & P \end{array}\right]}^{T}=0$$

Using the random sampling consensus algorithm to fit the plane, the ground model is determined as follows:

(5) $$X_{0}x+Y_{0}y+Z_{0}z=P_{0}$$

After obtaining the ground model, the ground information is removed from the point cloud data obtained by the depth camera, and the point cloud of obstacles is obtained. Then, the distance from each point to the plane is obtained by calculating Equation (6).

(6) $$d_{i}=\frac{\left|X_{0}x+Y_{0}y+Z_{0}z-P_{0}\right|}{\sqrt{X_{0}^{2}+Y_{0}^{2}+Z_{0}^{2}}}$$

The distance set $D_{S}\left\{d_{i}\right\}$ is obtained from all points of the obstacle to the plane, and then, the minimum bounding rectangle that envelops the obstacle in the point cloud is calculated based on all distances. The length, width, and height of the obstacle are calculated based on the 8 points of the minimum rectangle.

### 2.2. Gait Parameters Based on Obstacle Feature Information

Based on the obstacle information detected by the depth camera, the length, width, and height of the obstacle are obtained, as well as the distance between the obstacle and the lower limb exoskeleton robot, as shown in Equation (7).

(7) $$R=\varphi \left(I,x\right)$$

where I is the depth image information obtained by the depth camera; x contains status information of lower limb exoskeleton robots; and R includes the obstacle feature information $h_{o}$, $l_{o}$ and $w_{o}$, detected by depth cameras, and the distance $d_{o}^{r}$ between obstacles and lower limb exoskeleton robots.

Then, combined with the motion constraints of the lower limb exoskeleton robot, the required step length and step height for the lower limb exoskeleton robot to cross obstacles are obtained, as shown in Equation (8).

(8) $$\left(h_{a},d_{a}\right)=\xi \left[R\left(h_{o},l_{o},w_{o},d_{o}^{r}\right),C\right]$$

where $h_{a}$ and $d_{a}$ are the step height and step length when crossing obstacles, respectively. $\xi \left[\cdot \right]$ represents obtaining the appropriate step height and step length to cross the obstacle based on the feature information *R* of the obstacle and the motion constraint *C* of the lower limb exoskeleton robot.

### 2.3. Gait Trajectory Planning Based on Obstacle Feature Information

- Description of gait trajectory planning problem

Based on the periodicity of gait motions in lower limb exoskeleton robots, the dynamic system is constrained to a single support phase and simplified as a single-segment continuous trajectory optimization problem, with the system dynamics constrained as follows:

(9) $$\dot{x}=f\left(x,\tau_{a}\right)$$

where x is the state variable, and $\tau_{a}$ is the actual input torque.

The impact mapping of the end of the swinging leg colliding with the ground at the end of the gait is constrained as follows:

(10) $$x\left(0\right)=\Delta x\left(t_{F}\right)$$

where $t_{F}$ is the end time of a single gait.

The movements of lower limb exoskeleton robots cannot violate the physiological constraints of human joints. The knee joint movement angle is constrained.

(11) $$\theta_{t1}-\theta_{t2}\leq -\theta_{K\max}$$

where $\theta_{K\max}$ is the protective margin for excessive extension of the knee joint.

While walking, the human body has a slight inclination angle between the upper body and torso to ensure balance. The constraint on the forward and backward inclination angle of the torso is defined as

(12) $$\left\{\begin{array}{c} \theta_{t3}\left(t\right)\leq \theta_{to\text{-}f}\\ \theta_{t4}\left(t\right)\leq \theta_{to\text{-}b} \end{array}\right.$$

where $\theta_{to\text{-}b}$ and $\theta_{to\text{-}f}$ are the backward and forward inclination constraint of the torso.

To ensure that the motion trajectory is tracked by the motor, the upper limit of the input torque during the optimization process is determined based on the peak torque parameters of the joint motor of the lower limb exoskeleton robot, as shown in Equation (13).

(13) $$-\tau_{a\max}\leq \tau_{a}\left(t\right)\leq \tau_{a\max}$$

The constraint between the gait cycle time and step length can be expressed as

(14) $$P_{E}\left(t_{F}\right)={\left[\begin{array}{ccc} d_{a} & 0 & 0 \end{array}\right]}^{T}$$

where $P_{E}\left(t_{F}\right)$ is the position of the end of the swinging leg at the end of the gait cycle.

During gait movement, the end of the swinging leg should be higher than the ground. To avoid conflicts with the constraint that the end height of the swinging leg at the beginning and end of gait is zero, the ground clearance constraint is defined as

(15) $$P_{E\text{-}v}\left(t\right)\geq H\left(P_{E\text{-}h}\left(t\right)\right)$$

where $H\left(\cdot \right)$ is the ground clearance constraint function. $P_{E\text{-}v}\left(t\right)$ and $P_{E\text{-}h}\left(t\right)$ are the vertical and horizontal components of the swinging leg end $P_{E}$, respectively.

The constraint between the height of the swinging leg end and the gait cycle can be expressed as

(16) $$h_{t}=h_{a}-h_{a}\cdot \frac{{\left(t-\frac{t_{end}}{2}\right)}^{2}}{{\left(\frac{t_{end}}{2}\right)}^{2}}$$

where $h_{t}$ is the minimum height of the swinging leg end for each timestamp, the height constraint of the swinging leg end is $h_{t}-h_{op}<0$, and $h_{op}$ is the trajectory of the height of the swinging leg end that needs to be optimized and solved. $h_{a}$ is the expected height for lifting the leg. $t_{end}$ is the end of a gait cycle time.

The objective function is defined as the time integral of the quadratic function of the input torque:

(17) $$J=\int_{0}^{T}\tau_{a}^{T}\cdot Z\cdot \tau_{a}dt$$

where Z is a quadratic matrix.

The purpose of the objective function is to prevent excessive torque locally, where the quadratic form is a positive definite matrix. If it is a semi-positive definite matrix, this will result in an input torque that maintains the upper limit value in the optimization result. A positive definite matrix can smooth the curves of an input torque and state variables, reducing errors in approximating the state trajectory using smooth curves [29].

The above constraints and objective function are summarized as a trajectory optimization problem:

(18) $$\begin{array}{ll} \min_{t_{0},t_{F},x\left(t\right),\tau_{a}\left(t\right)} & J=\int_{0}^{T}\tau_{a}^{T}\cdot Z\cdot \tau_{a}dt\\ s.t. & \dot{x}=f\left(x,\tau_{a}\right)\\ & x\left(0\right)=\Delta x\left(t_{F}\right)\\ & \theta_{t1}(t)-\theta_{t2}(t)\leq -\theta_{K\max}\\ & \theta_{t3}(t)\leq \theta_{to\text{-}f}\\ & \theta_{t4}(t)\leq \theta_{to\text{-}b}\\ & -\tau_{a\max}\leq \tau_{a}\left(t\right)\leq \tau_{a\max}\\ & P_{E,k}\left(t_{F}\right)={\left[\begin{array}{ccc} d_{a} & 0 & 0 \end{array}\right]}^{T}\\ & h_{t}=h_{a}-h_{a}\cdot \left({\left(t-\frac{t_{end}}{2}\right)}^{2}/{\left(\frac{t_{end}}{2}\right)}^{2}\right)\\ & h_{t}-h_{op}<0\\ & P_{E\text{-}v,k}\left(t\right)\geq H\left(P_{E\text{-}h,k}\left(t\right)\right) \end{array}$$

- Offline gait trajectory planning

The trajectory planning problem belongs to the optimal control problem. The direct collocation method applied to trajectory planning problems discretizes the continuous optimal control process, takes the obtained coefficients as new variables, and then uses modern optimization tools to solve nonlinear optimization problems [30,31].

Continuous functions are related to state variables and control variables and require interpolation approximations based on discrete points for state variables and control variables. The target optimization trajectory is divided into *N* interpolation segments (including starting point, midpoint, and endpoint), and the endpoints of adjacent interpolation segments are the same as the starting point, so there are 2*N* + 1 sampling points, as shown in Equation (19).

(19) t0,⋯,tk,tk+12,tk+1,⋯,tN

The time for each interpolation segment is

(20) $$\zeta_{k}=t_{k+1}-t_{k}$$

Similarly, the state variables and control variables are also divided into 2*N* + 1 sampling points:

(21) x0,⋯,xk,xk+12,xk+1,⋯,xNτ0,⋯,τk,τk+12,τk+1,⋯,τN

Sampling points are used to define interpolation functions to approximate the original function. The integral of a quadratic interpolation function can be represented by the function values of three points (starting point, midpoint, and endpoint) in the integration interval. Assuming the quadratic function $\gamma \left(t\right)$

(22) $$\gamma \left(t\right)=a+bt+ct^{2}$$

where, *a*, *b*, and *c* are constants.

The starting point, midpoint, and endpoint of the quadratic interpolation function in the interval $\left[0,\zeta \right]$ can be expressed as

(23) γ0=aγζ2=a+ζ2b+ζ24cγζ=a+ζb+ζ2c

Therefore, from (23),

(24) a=γ0bζ=−3γ0+4γζ2−rγζcζ2=2γ0−4γζ2+2γζ

The integral *U* of the quadratic function $\gamma \left(t\right)$ on the interval $\left[0,\zeta \right]$ can be expressed as

(25) U=∫0ha+bt+ct2=aζ+12bζ2+13cζ3=hζ6γ0+4γζ2+γζ

According to (25), the integral of the derivative of the system state between two nodes in a single interpolation segment can be expressed as

(26) $$x_{k+1}-x_{k}=\int_{t_{k}}^{t_{k+1}}\dot{x}dt=\int_{t_{k}}^{t_{k+1}}f\left(x,\tau_{a}\right)dt$$

where $f\left(\cdot \right)$ represents the system dynamic equation, and $x_{k}$ represents the system state at the *k*-th node.

Equation (26) can be written as

(27) xk+1−xk=ζk6fxk,τak+4fxk+12,τak+12+fxk+1,τak+1

The left side of Formula (27) represents the difference between the starting and ending states of a single interval, while the right side represents the estimated integral value of the derivative of the state variables at three points within the interval. In the nonlinear optimization solution, it can be defined as an equation relationship. The variable xk+12, to be optimized in Formula (27), satisfies the constraint relationship with the starting and ending points of the interval:

(28) xk+12=12xk,xk+1+ζk8fxk,τak−fxk+1,τak+1

Equations (27) and (28) are system dynamic constraints based on discrete variables.

Similarly, a similar approach can be applied to the objective function. The integrand of the objective function can be expressed as

(29) $$l\left(t\right)=\tau_{a}{\left(t\right)}^{T}\cdot Z\cdot \tau_{a}\left(t\right)$$

The integral within the gait cycle can be expressed as

(30) ∫t0tFltdt≈∑k=0N−1hk6lk+4lk+12+lk+1

Therefore, the objective function can be expressed as

(31) J=∑k=0N−1hk6lk+4lk+12+lk+1

Ultimately, the nonlinear optimization problem of the transformed trajectory planning can be summarized as

(32) mint0,tF,xt,τatJ=∑k=0N−1hk6lk+4lk+12+lk+1s.t.xk+1−xk=ζk6fxk,τak+4fxk+12,τak+12+fxk+1,τak+1x˙=fx,τax0=ΔxtFθt1(t)−θt2(t)≤−θKmaxθt3(t)≤θto-fθt4(t)≤θto-b−τamax≤τa1,a2,a3,a4,k(t)≤τamaxPE,ktF=da00Tht=ha−ha⋅t−tend22/tend22ht−hop<0PE-v,kt≥HPE-h,kt

The optimized discrete state variables and control variables are interpolated to obtain the desired continuous trajectory. The local time variables within the interpolation segment are specified as

(33) $$\kappa =t-t_{k}$$

Therefore, the interpolation reconstruction of the control trajectory is represented as

(34) τat=2ζk2τak⋅κ−ζkκ−ζk2−2τak+12⋅τκτκ−ζk+τak+1⋅κκ−ζk2

The dynamic equation is reconstructed as

(35) x˙t=fxt,τat=2ζk2fxk,τak⋅κ−ζkκ−ζk2−fxk+12,τak+12⋅2κκ−ζk+fxk+1,τak+1⋅κκ−ζk2

By integrating the dynamic equations, the state variables are reconstructed as

(36) xt=∫0tx˙κdτ=xk+ζk⋅fxk,τak⋅κζk+12−3fxk,τak+4fxk+12,τak+12−fxk+1,τak+1⋅κζk2+132fxk,τak−4fxk+12,τak+12+2fxk+1,τak+1⋅κζk3

According to the nonlinear optimization results, the optimized motion trajectory can be obtained by setting the expected step length, step height, and cycle time. The gait and motion constraint parameters defined for gait trajectory optimization are shown in Table 1.

During the assisted-movement process of lower limb exoskeleton robots, the feature information of obstacles (size information, such as height h and length $d_{o}$ of obstacles) and the distance between obstacles and lower limb exoskeleton robots are detected through depth cameras. Then, the lower limb exoskeleton robot control system calculates the required leg lift height $h_{a}$ and step length $d_{a}$ to cross the obstacle based on the obstacle size information, as shown in Equation (37):

(37) $$\left\{\begin{array}{l} h_{a}=h+\Delta h\\ d_{a}=d_{1}+d_{o}+d_{2} \end{array}\right.$$

In obstacle-avoidance movements based on obstacle detection information, there is the problem of being too close or too far from obstacles during normal gait movements. This paper uses optimization methods to set the distance between different obstacles and lower limb exoskeleton robots, as well as the feature information of obstacles, to generate offline adjusted gait trajectory libraries and obstacle-crossing gait trajectory libraries containing multiple gait parameters.

(1) Adjusted gait trajectory: when the distance between the lower limb exoskeleton robot and the obstacle cannot achieve safe crossing within one gait cycle, the step length needs to be adjusted appropriately when approaching the obstacle.
(2) Obstacle-crossing gait trajectory: when the lower limb exoskeleton robot reaches a safe crossing position after adjusting its gait trajectory, it selects an appropriate obstacle-crossing gait trajectory from the trajectory library based on the detected obstacle information.

It should be mentioned that when generating the trajectory library offline, the motion trajectory of the lower limb exoskeleton robot crossing obstacles considers the dynamics of the human–robot system. Therefore, the gait trajectory selected in both trajectory libraries is the optimal trajectory that can safely cross obstacles.

### 2.4. Obstacle-Crossing Motion Decision-Making Algorithm Based on Obstacle Feature Information

**Figure 1:**
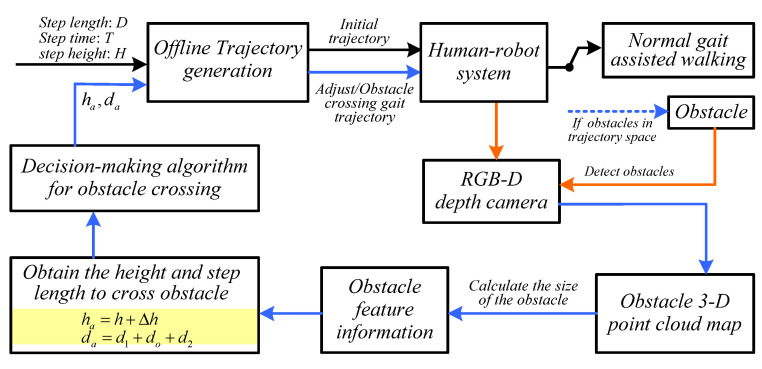
Obstacle-crossing-motion control framework.

The lower limb exoskeleton robot’s obstacle-crossing-motion control framework based on obstacle feature information is shown in Figure 1.

The lower limb exoskeleton robot mainly focuses on two tasks when crossing obstacles: (1) accurately detect obstacle feature information that affects assisted motions in the environment, and (2) select an appropriate motion trajectory based on the detected obstacle feature information to achieve safe and accurate obstacle-crossing motion. To achieve safe obstacle-crossing movements of lower limb exoskeleton robots, it is necessary to construct a decision-making algorithm for obstacle-crossing movements of lower limb exoskeleton robots based on obstacle feature information, as shown in Algorithm 1. The obstacle-crossing motion decision-making algorithm selects the appropriate gait trajectory from two trajectory libraries based on gait constraints, motion constraints, obstacle feature information, and the distance between the obstacle and the lower limb exoskeleton robot, allowing the lower limb exoskeleton robot to safely cross obstacles.
**Algorithm 1.** Decision-making algorithm for obstacle-crossing motions of lower limb exoskeleton robot**Input:** Gait information: step length $d_{a}^{*}$, step height $h_{a}^{*}$, step time $T^{*}$;   Safety constraints: protective margin for excessive extension of the knee joint $\theta_{K\max}$, constraint on the forward and backward inclination angle of the torso $\theta_{to\text{-}f}$ and $\theta_{to\text{-}b}$   Other parameters: $d_{s}$, $d_{1}$, $d_{2}$, and $d_{f}$**Output: If** no obstacles are detected, execute the normal motion trajectory based on the initial gait information;   Enter initial gait information.   Enter a normal gait trajectory with a step length of $d_{a}^{*}$, a step height of $h_{a}^{*}$, and a gait cycle of $T^{*}$;**Repeat**   **Else**   **If** obstacles in the motion trajectory space are detected, obtain obstacle feature information and the distance between the obstacle and the lower limb exoskeleton robot, and execute the obstacle-crossing-motion decision-making algorithm;     **If** the distance $d_{c}$ between the obstacle and the lower limb exoskeleton robot meets condition $d_{a}^{*}<d_{c}<1.5d_{a}^{*}$, based on the detected distance, the robot controller adjusts the gait trajectory library to a trajectory that is less than $d_{a}^{*}$ but greater than 0.5$d_{a}^{*}$ and moves it for one motion cycle. If the height d of the obstacle is less than or equal to the set threshold $d_{s}$ ($d\leq d_{s}$), the lower limb exoskeleton robot executes a normal motion trajectory.    **Else**     **If** $d>d_{s}$, the depth camera detects spatial feature information of obstacles: h, $d_{o}$ and $d_{c}$.      **If** $d_{c}$ is greater than the set threshold $d_{1}$, and $d_{c}-d_{a}-d_{f}>0$, execute the normal trajectory.      **Else** calculate the step height $h_{a}$ and step length $d_{a}$ ($h_{a}=h+\Delta h$; $d_{a}=d_{1}+d_{o}+d_{2}$) for safely crossing obstacles based on obstacle feature information. The robot controller selects the appropriate trajectory from the obstacle-crossing gait trajectory library based on the calculated $h_{a}$ and $d_{a}$ to complete the crossing motion. And then, execute the normal motion trajectory.      **End if**     **End if **    **End if****Until** Gait movement ends

The obstacle-crossing-motion decision-making algorithm includes three gait motion modes, normal motions, transitional motions, and obstacle-crossing motions, which are used for switching the motion trajectory of lower limb exoskeleton robots to ensure the safety of motions. The three gait motion modes are as follows:

(1) Normal motions: the lower limb exoskeleton robot maintains a fixed step length and leg lift height in its motion trajectory.
(2) Transition motions: the gait distance is adjusted based on the distance information between the detected lower limb exoskeleton robot and obstacles, so that the lower limb exoskeleton robot moves to the appropriate position, ensuring the safety of obstacle-crossing motions.
(3) Obstacles-crossing motions: the lower limb exoskeleton robot detects obstacle feature information through a depth camera, adjusts the distance through transition motions, selects appropriate crossing motion trajectory, and safely crosses obstacles.

## 3. Experimental Section

**Figure 2:**
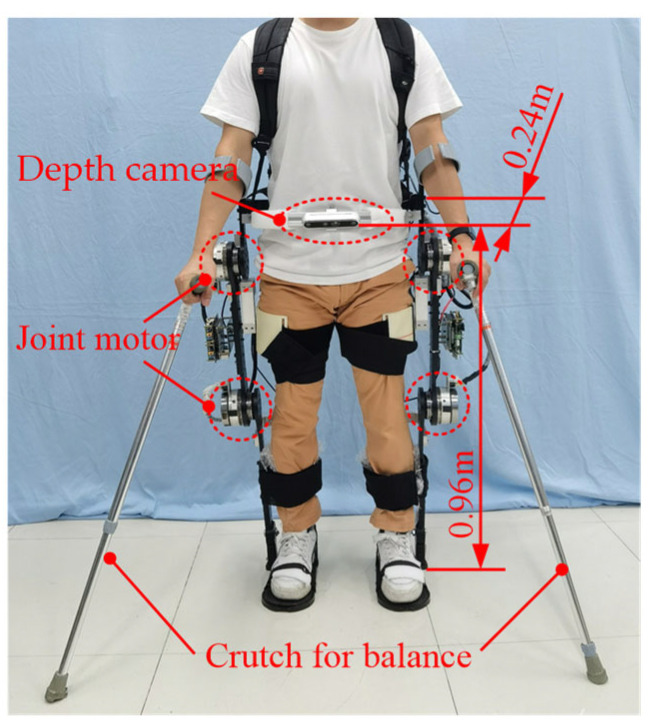
Lower limb exoskeleton robots integrated with depth cameras.

This experiment was conducted with the assistance of three volunteers, including one wearing a lower limb exoskeleton robot, one assisting beside the wearer to prevent falls, and one responsible for the start and stop switches of the lower limb exoskeleton robot. Volunteers wore lower limb exoskeleton robots integrated with depth cameras to conduct obstacle-crossing-motion experiments, as shown in Figure 2.

The depth camera was installed in the middle of an aluminum bracket through a threaded hole, and the aluminum bracket was fixed on both sides of the waist link of the lower limb exoskeleton robot. The vertical height of the depth camera to the ground was 0.96 m, and the horizontal distance from the lower limb exoskeleton robot’s waist link was 0.24 m. The depth camera communicated with a high-performance computer through a USB transmission cable and transmitted information to the controller of the lower limb exoskeleton robot.

### 3.1. Experimental Design for Different Obstacle Distances

To verify the proposed obstacle feature information-based lower limb exoskeleton robot obstacle-crossing-motion decision-making algorithm, three experiments were conducted to determine the distances between obstacles and lower limb exoskeleton robots. The experimental design was as follows:

(1) To ensure that the obstacle feature information detected by the depth camera was the same, the lower limb exoskeleton robot was set to cross the same obstacles.
(2) The motion step length of the lower limb exoskeleton robot was 0.28 m, and the initial distance between the obstacle and the lower limb exoskeleton robot must have been greater than two step lengths. In the experiment, we set the step time to 1.6 s.
(3) Due to measurement errors between obstacles and robots, the measured distance was rounded to two decimal places. The distance between the obstacle and the robot was set to 1.26 m, 1.14 m, and 1.21 m, respectively.
(4) To ensure that there was no sudden change in position during movements, the starting state of each gait trajectory was consistent with the ending state of the previous trajectory. In two types of offline trajectory library generations, this situation was considered.

### 3.2. Experimental Design of Different Obstacle Feature Information

Four types of obstacles with different feature information were designed as objects for the lower limb exoskeleton robot to cross to verify the algorithm proposed in this paper. The actual size and measure size of the obstacles are shown in Table 2. Table 2 shows that the measure size of the four obstacles was larger than the actual size. The reason for this is that when depth cameras detect obstacle feature information, computer vision algorithms use 3D point cloud filtering and downsampling methods to generate and display bounding boxes that can surround the contours of obstacles, resulting in measurement errors. It should be noted that this paper focuses on trajectory planning and obstacle-crossing motions based on obstacle feature information. Therefore, the small measurement error caused by obstacle detection is within an acceptable range. The 3D point cloud was projected onto a 2D image to mark bounding boxes on the original image, and the feature information of the detected obstacles, including height, width, and length, were calculated. At the same time, the depth sensor embedded in the depth camera detected the distance between the lower limb exoskeleton robot and obstacles.

## 4. Results and Discussion

### 4.1. Experimental Results for Motion Decision-Making for Different Obstacle Distances

**Figure 3:**
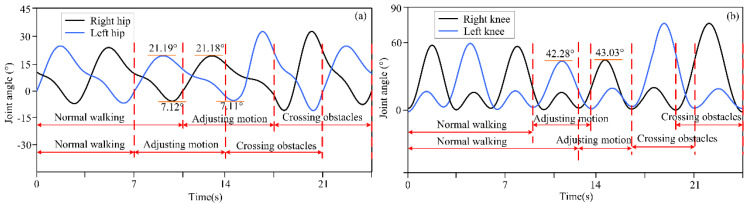
(**a**) The motion trajectory of hip joint at a distance of 1.26 m between obstacles and lower limb exoskeleton robots; (**b**) The motion trajectory of knee joint at a distance of 1.26 m between obstacles and lower limb exoskeleton robots.

**Figure 4:**
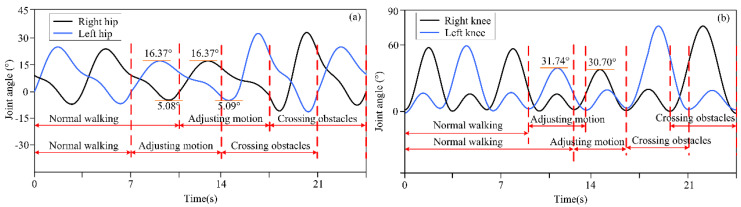
(**a**) The motion trajectory of hip joint at a distance of 1.14 m between obstacles and lower limb exoskeleton robots; (**b**) The motion trajectory of knee joint at a distance of 1.14 m between obstacles and lower limb exoskeleton robots.

**Figure 5:**
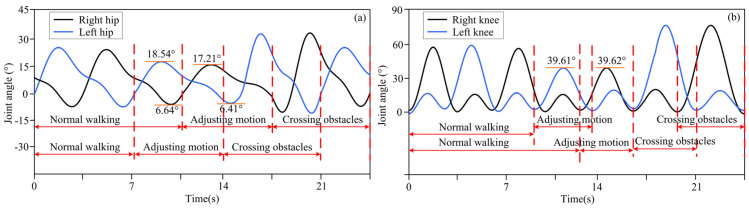
(**a**) The motion trajectory of hip joint at a distance of 1.21 m between obstacles and lower limb exoskeleton robots; (**b**) The motion trajectory of knee joint at a distance of 1.21 m between obstacles and lower limb exoskeleton robots.

The actual distance between different obstacles and the lower limb exoskeleton robot was set as described above, and the distance information was detected by a depth camera and transmitted to the lower limb exoskeleton robot controller. Based on decision-making algorithms, the controller selected an appropriate gait trajectory from the adjusted gait trajectory library based on the detected distance information to complete gait motion adjustments. The experimental results of the lower limb exoskeleton robot at three different distances from obstacles are shown in Figure 3, Figure 4 and Figure 5. Figure 3, Figure 4 and Figure 5 show the motion trajectory curves of the hip and knee joints at obstacle distances of 1.26 m, 1.14 m, and 1.21 m, respectively.

From the experimental results of different distances between lower limb exoskeleton robots and obstacles, it can be seen that by the proposed decision-making algorithm, lower limb exoskeleton robots can select appropriate gait trajectories and adjust their movements based on distance information. Based on the distance information between obstacles and the robot detected by the depth camera, the lower limb exoskeleton robot executes a normal gait trajectory when the distance from the obstacle is far. When the lower limb exoskeleton robot approaches an obstacle and determines that it will collide with the obstacle with a normal trajectory, the lower limb exoskeleton robot will switch to adjusting the trajectory. The controller selects an appropriate gait adjustment trajectory from the trajectory library, and the joint angles of the hip and knee joints of the gait adjustment trajectory decrease. At this time, the corresponding step length of the lower limb exoskeleton robot decreases. The hip and knee joint angles of gait adjustment trajectories for obstacles and lower limb exoskeleton robots at different distances are shown in Table 3. After executing the gait adjustment trajectory, the lower limb exoskeleton robot approaches the obstacle and switches to the obstacle-crossing gait trajectory in the next gait. At this point, the lower limb exoskeleton robot controller will select an appropriate trajectory from the obstacle-crossing gait trajectory library based on the feature information of the obstacle for the obstacle-crossing motion.

### 4.2. Obstacle-Crossing Experiment with Different Obstacle Feature Information

**Figure 6:**
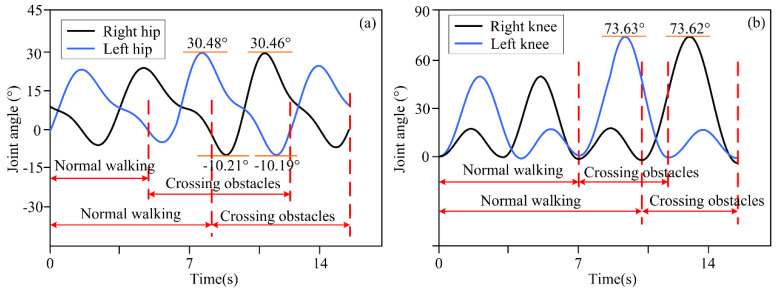
(**a**) Joint angles of hip joint crossing the trajectory of obstacle 1; (**b**) Joint angles of knee joint crossing the trajectory of obstacle 1.

**Figure 7:**
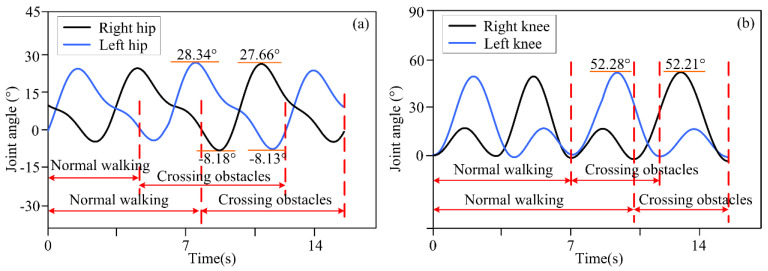
(**a**) Joint angles of hip joint crossing the trajectory of obstacle 2; (**b**) Joint angles of knee joint crossing the trajectory of obstacle 2.

**Figure 8:**
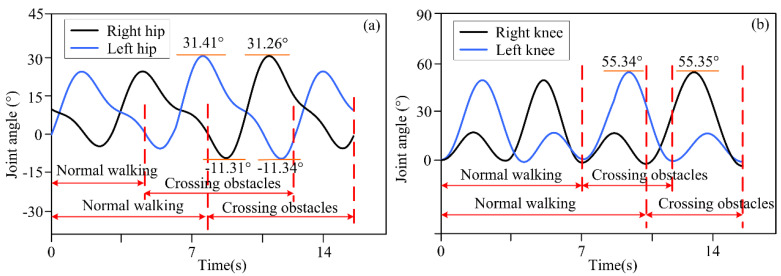
(**a**) Joint angles of hip joint crossing the trajectory of obstacle 3; (**b**) Joint angles of knee joint crossing the trajectory of obstacle 3.

**Figure 9:**
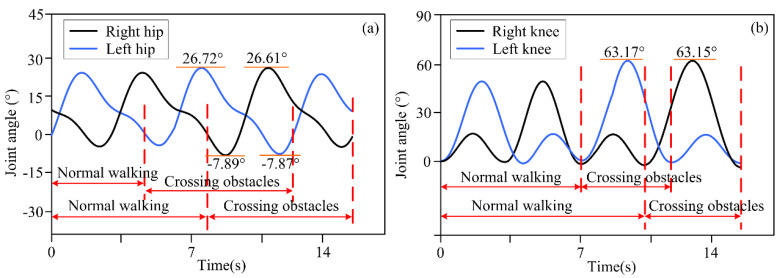
(**a**) Joint angles of hip joint crossing the trajectory of obstacle 4; (**b**) Joint angles of knee joint crossing the trajectory of obstacle 4.

Volunteers wore the lower limb exoskeleton robot for four obstacle-crossing experiments, and the joint motion angles are shown in Figure 6, Figure 7, Figure 8 and Figure 9. In terms of Figure 6, Figure 7, Figure 8 and Figure 9, it can be seen that for different obstacles, the lower limb exoskeleton robot will select appropriate gait trajectories from the obstacle-crossing gait trajectory library based on the obstacle feature information collected by the depth camera to complete safe obstacle-crossing movements. The angles of the hip and knee joints of the lower limb exoskeleton robot corresponding to the crossing motion of different obstacles are different, which is related to the gait trajectory corresponding to the characteristic parameters of the obstacles. Based on the obstacle feature information, when the height and length of the obstacle increase, the lower limb exoskeleton robot will choose an appropriate motion trajectory based on motion and gait constraints to ensure safe crossing. The corresponding hip and knee joint motion angles will be greater than the joint angles under a normal gait trajectory. When the height of the obstacle is small, but the length is large, the increase in the hip joint angle crossing the gait trajectory is large, but the increase in the knee joint angle is small.

**Figure 10:**
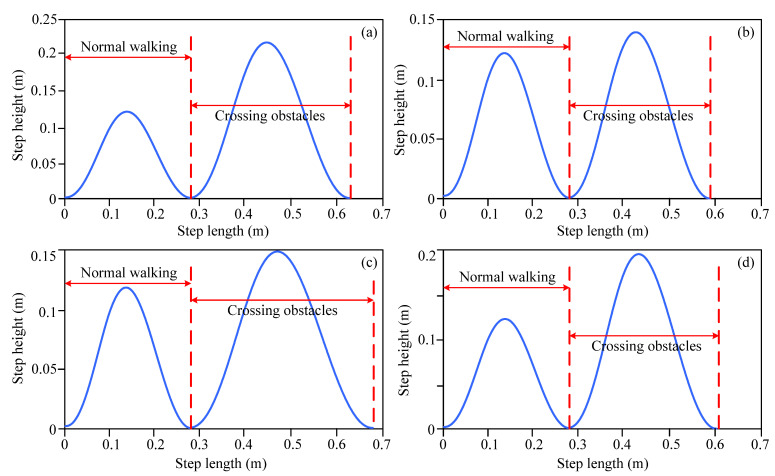
Step length and step height of lower limb exoskeleton robot when obstacle-crossing. (**a**) The step length and step height of obstacle-crossing 1; (**b**) The step length and step height of obstacle-crossing 2; (**c**) The step length and step height of obstacle-crossing 3; (**d**) The step length and step height of obstacle-crossing 4.

Figure 10 shows the spatial trajectory curves of the step length and step height corresponding to the end of the swinging leg of the lower limb exoskeleton robot when crossing obstacles 1, 2, 3, and 4. In terms of Figure 10, it can be seen that the spatial trajectory curve of the end of the lower limb exoskeleton robot when crossing obstacles follows an approximate sine trajectory, which is consistent with the characteristics of a foot spatial trajectory during human gait movements. When crossing obstacles, the spatial trajectory of the end of the lower limb exoskeleton robot changes accordingly, and the step length and step height are adjusted accordingly. Table 4 shows the specific values of gait parameters, such as the step height and step length, when the end of the swinging leg of the lower limb exoskeleton robot crosses obstacles with different feature information.

By analyzing the joint motion angles and corresponding spatial trajectory curve changes when crossing different obstacles, from Figure 3, Figure 4, Figure 5, Figure 6, Figure 7, Figure 8, Figure 9 and Figure 10, it can be observed that the decision-making algorithm for obstacle-crossing proposed in this paper for lower limb exoskeleton robots based on obstacle detection information is effective. Through this decision-making algorithm, an appropriate adjusted gait trajectory and crossing gait trajectory can be selected based on the distance between the obstacles and the robot and obstacle feature information to ensure safe obstacle-crossing movements of the lower limb exoskeleton robot and improve its environmental adaptability.

### 4.3. Limitations and Future Work

This paper introduces computer vision technology into lower limb exoskeleton robots. Through gait trajectory planning methods, two gait trajectory libraries were generated offline based on different obstacle feature information and different distances between obstacles and lower limb exoskeleton robots. During the motion process, computer vision technology is used to detect obstacle features and distances and to select appropriate trajectories from the trajectory library, which belongs to offline motion planning methods.

In future work, online gait trajectory planning based on environmental information will be considered. By using the feature information of obstacles and the distance information between obstacles and lower limb exoskeleton robots, scientific obstacle-crossing motion trajectories will be generated in real time to drive lower limb exoskeleton robots to achieve obstacle-crossing movements. This approach is more in line with daily motion environments and usage needs. At the same time, experiments will also be considered in darker environments and with different obstacles, people, walking speeds, and pathological gaits, and the adjustment ability of lower limb exoskeleton robots will be evaluated.

However, the online gait trajectory planning of lower limb exoskeleton robots faces more complex environments and obstacles with complex appearance features, which require higher requirements for obstacle detection algorithms and computer hardware systems for model computation.

## 5. Conclusions

This paper combines computer vision technology with lower limb exoskeleton robots to study the method of obstacle-crossing for lower limb exoskeleton robots. Feature information of obstacles is obtained in the motion environment of lower limb exoskeleton robots and the distance between lower limb exoskeleton robots and obstacles by depth cameras. The gait planning method, based on the direct collocation method, generates an offline adjusted gait trajectory library and an obstacle-crossing gait trajectory library containing multiple sets of gait parameter information. A lower limb exoskeleton robot obstacle-crossingmotion decision-making algorithm based on obstacle feature information was proposed by combining gait and motion constraints. Appropriate motion trajectories are selected from the trajectory library based on the detected obstacle feature information to achieve safe obstacle-crossing motions for lower limb exoskeleton robots. Obstacle-crossing experiments were conducted with three distances between obstacles and robots, as well as obstacle-crossing experiments with feature information from four obstacles. The experimental results show that the proposed method can enable lower limb exoskeleton robots to select appropriate trajectories in the trajectory library, adjust gait movements, and safely cross obstacles. The step height and step length of the four motion trajectories when crossing four obstacles were 0.22 m and 0.34 m, 0.14 m and 0.31 m, 0.15 m and 0.40 m, and 0.19 m and 0.33 m, respectively.

## Figures and Tables

**Table 1:** Parameters of gait constraints and motion constraints defined in gait trajectory planning.

Parameter Symbols	Parameter Meaning	Values
$\theta_{to\text{-}b}$	Backward inclination angle of the torso	$-0.03\left(rad\right)$
$\theta_{to\text{-}f}$	Forward inclination angle of the torso	$0.03\left(rad\right)$
$\theta_{K\max}$	Protective margin for excessive extension of the knee joint	$0.03\left(rad\right)$
$d_{a}$	Step length when crossing obstacles	Calculated from the length of obstacles
$h_{a}$	Step height when crossing obstacles	Calculated from the height of obstacles
$H\left(h_{a}\right)$	Ground clearance constraint for swinging legs	$H\left(h_{a}\right)=-0.024{\left(h_{a}\right)}^{2}+0.024$
Δh	Safety margin for crossing obstacles	Customized according to experimental constraints
$T^{*}$	Step time	Set up from the experiment
$d_{a}^{*}$	One step length of normal trajectory	From the normal trajectory measurement of the experiment
$h_{a}^{*}$	Step height of normal trajectory	From the normal trajectory measurement of the experiment
$d_{s}$	Threshold for trajectory selection in motion decision-making algorithm for obstacle crossing	Customized from experimental constraints
$d_{c}$	Actual horizontal distance between the support foot and the obstacle	Obtained from depth camera detection
$d_{1}$	Horizontal distance between the support foot and the obstacle	Customized from experimental constraints
$d_{2}$	Horizontal distance between the swinging foot and the obstacle after landing	Customized from experimental constraints
$d_{f}$	Horizontal distance extending from the toe to the end of the swinging leg	Customized from experimental constraints

**Table 2:** Actual size and measure size of four types of obstacles.

Real Images	Size	Length (m)	Width (m)	Height (m)
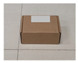	Actual size	0.125	0.019	0.08
	Measure size	0.1284	0.19702	0.0921
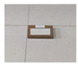	Actual size	0.11	0.20	0.03
	Measure size	0.1307	0.2063	0.0386
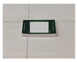	Actual size	0.185	0.26	0.03
	Measure size	0.1901	0.2704	0.4011
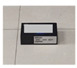	Actual size	0.09	0.165	0.053
	Measure size	0.1012	0.1723	0.6029

**Table 3:** Each joint angle of adjustment trajectory at different distances of obstacle and robot.

Distances	Joint Angles (°)			
	Left Hip	Left Knee	Right Hip	Right Knee
Distance 1: 1.26 m	21.19	42.28	21.18	43.03
	7.12		7.11	
Distance 2: 1.14 m	16.37	31.74	16.37	30.70
	5.08		5.09	
Distance 3: 1.21 m	18.54	39.61	17.21	39.62
	6.64		6.41	

**Table 4:** Specific values of gait parameters of lower limb exoskeleton robots crossing obstacles with different feature information.

Objects	Specific Values	
	Step Height (m)	Step Length (m)
Obstacle 1	0.22	0.34
Obstacle 2	0.14	0.31
Obstacle 3	0.15	0.40
Obstacle 4	0.19	0.33

## Data Availability

The original contributions presented in this study are included in the article.
